# Optical Quantification of Metal Ions Using Plasmonic Nanostructured Microbeads Coated with Metal–Organic Frameworks and Ion-Selective Dyes

**DOI:** 10.1021/acsnanoscienceau.2c00063

**Published:** 2023-03-06

**Authors:** Tolga Zorlu, Begoña Puértolas, I. Brian Becerril-Castro, Luca Guerrini, Vincenzo Giannini, Miguel A. Correa-Duarte, Ramon A. Alvarez-Puebla

**Affiliations:** † Department of Physical and Inorganic Chemistry, Universitat Rovira i Virgili, Carrer de Marcel·lí Domingo s/n, 43007 Tarragona, Spain; ‡ Department of Physical Chemistry, Center for Biomedical Research (CINBIO), Southern Galicia Institute of Health Research (IISGS) and Biomedical Research Networking Center for Mental Health (CIBERSAM), Universidade de Vigo, 36310 Vigo, Spain; § Technology Innovation Institute, Masdar City, 9639 Abu Dhabi, United Arab Emirates; ∥ Centre of Excellence ENSEMBLE3 sp. z o.o., Wolczynska 133, 01-919 Warsaw, Poland; ⊥ ICREA, Passeig Lluís Companys 23, 08010 Barcelona, Spain

**Keywords:** SERS, plasmonic microbeads, metal−organic frameworks, metallic ions, ion-selective dyes

## Abstract

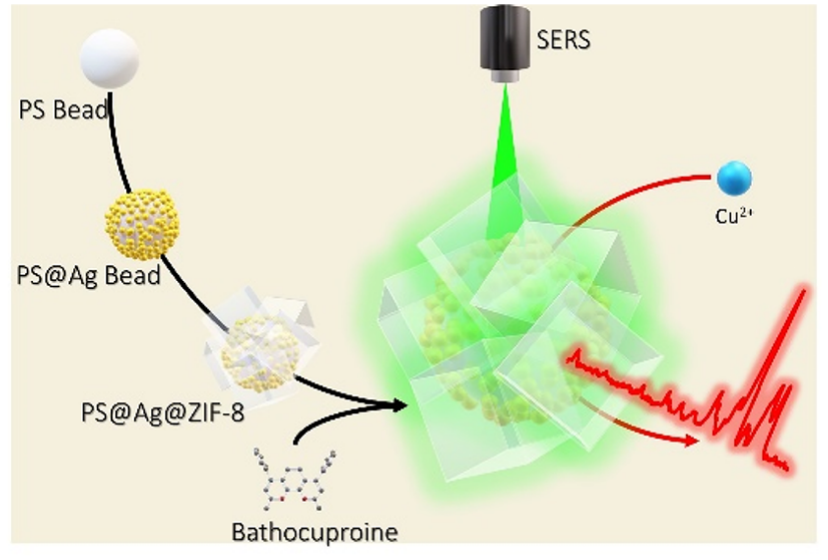

Herein, we designed and synthesized a hybrid material comprising polystyrene submicrobeads coated with silver nanospheres. This material provides a dense collection of electromagnetic hot spots upon illumination with visible light. The subsequent coating with a metal-framework and the adsorption of bathocuproine on it yield an optical sensor for SERS that can specifically detect Cu(II) in a variety of aqueous samples at the ultratrace level. Detection limits with this method are superior to those of induced coupled plasma or atomic absorption and comparable with those obtained with induced coupled plasma coupled with a mass detector.

## Introduction

Surface-enhanced Raman scattering (SERS) is a powerful ultrasensitive technique based on the close contact of the target analyte with a plasmonic particle.^1−3^ However, as a molecular spectroscopy, SERS is unable to detect atomic species.^4^ These species, and particularly metallic ions, represent a good portion of water and soil pollution^5^ and also have key interest in the study of biological processes^6^ or even the diagnosis of certain diseases.^7^ Thus, to apply this sensitive analytical technique to the detection of metals (and other atomic ions), usually plasmonic surfaces are functionalized with molecules (i.e., chemosensors) that react with these species.^4^ In such a case, the atomic analyte is indirectly detected through the changes that it induces on the molecular^8^ or electronic^9^ structure or in the orientation of the chemosensor.^10^ Notably, although the classic analytical literature describing the use of organic reagents specific for each metal ion, and sometimes each oxidation state for the same ion, is extensive,^11−14^ the use of such molecules in SERS is very restricted because of the necessity of the chemosensor to be explicitly attached to the plasmonic surface. In such conditions, the most common chemosensors for atomic ion analysis rely on the use of thiolated small aromatic molecules that are functionalized metal reactive moieties such as carboxylic, amino, or alcohol groups that can react with the target metals.^15^ This strategy, however, shows little specificity and, while it can be successfully applied to simple analytical problems, does not work for complex fluids such as those of biological or natural origin.

Metal–organic frameworks (MOFs) are porous materials with homogeneous pores formed by the coordination of organic linkers with metal ions.^16,17^ During the past two decades, MOFs have demonstrated utility in a diversity of applications ranging from catalysis, separation, drug delivery, or analytical chemistry, either alone or in the form of composites with other materials.^18−20^ In particular, plasmonic nanoparticles coated with MOFs have been successfully applied to the analysis of small molecules,^21,22^ drug delivery,^23,24^ and pollutant separation.^25−28^ Commonly, these materials are prepared by coating single particles with a thin shell of MOF or by dispersing multiple but isolated particles within an MOF matrix. Either way, this is due to the lack of interaction between particles to form electromagnetic hot spots.^29^ Thus, to ensure the electromagnetic field is sufficient to produce a good SERS signal, these composites usually employ single particle hot spots such nanostars,^24,30^ a well-known plasmonic particle capable of localizing a strong electromagnetic field at their tips.^31^ These composite MOF–nanostar materials are very useful upon near-infrared illumination, but their localized surface plasmon resonance (LSPR) below 700 nm is modest, with a subsequent limited optical enhancement.^31^

Herein, we designed and synthesized a hybrid material comprising polystyrene submicrobeads coated with silver nanospheres. This material provides a dense collection of electromagnetic hot spots upon illumination with visible light.^9^ Subsequently, the plasmonic beads were coated with MOF providing a plasmonic material with properties that are the same as, or even better than, common nanostars coated with MOFs, but in the visible. Finally, to probe the efficiency of this composite for ultratrace analysis of ions, and exploiting the affinity of MOFs for nonpolar organic molecules, a selective dye for copper, bathocuproine,^32^ was adsorbed in the composite material. The sensing platform was then used for the analysis of copper ions in aqueous samples of different natural origin.

## Results and Discussion

**Figure 1:**
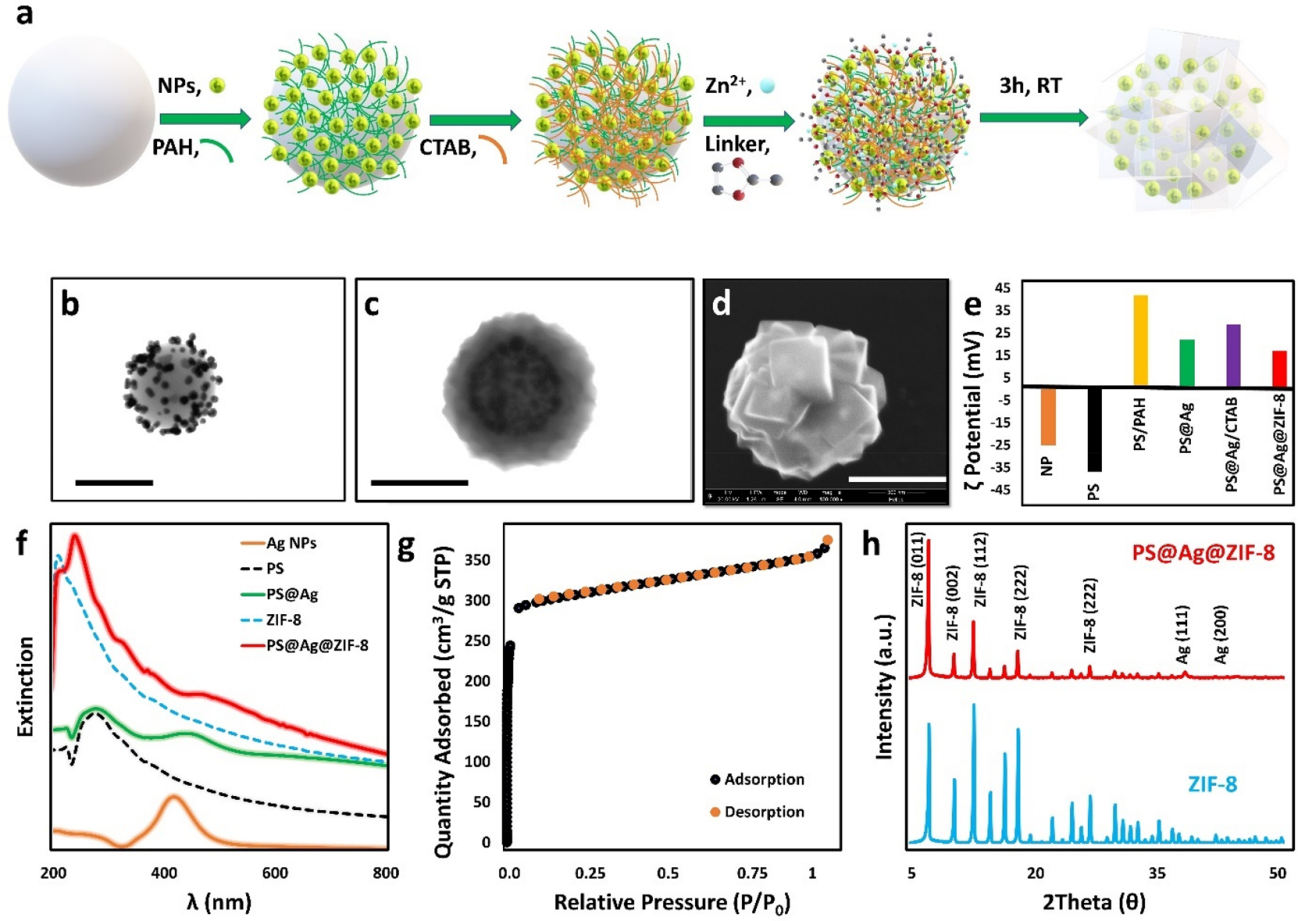
(a) Schematic illustration of preparation stages of PS@Ag@ZIF-8 SERS substrates. (b) Representative TEM images of PS@Ag beads. (c, d) Representative TEM and SEM images of the composites, respectively. Scale bars, 500 nm. (e) ζ potential at the different fabrication steps of the composite. The final particles have nearly +17 mV. (f) Extinction spectra of AgNPs, PS beads, PS@Ag beads, pristine ZIF-8, and PS@Ag@ZIF-8 suspensions. (g) Nitrogen adsorption–desorption isotherm of PS@Ag@ZIF-8 composite. (h) X-ray diffraction (XRD) patterns of PS@Ag@ZIF-8 and pristine ZIF-8 crystals.

The schematic illustration of the preparation steps of the Ps@Ag@ZIF-8 composites is given in Figure 1a. Accordingly, PS beads (496 ± 16 nm diameter) were coated with a layer of the positively charged polymer [poly(allylamine hydrochloride, PAH]. Subsequently, the material was exposed to an excess of spherical negatively charged silver nanospheres (AgNPs) of 51 ± 6 nm size (Figure S1) to promote their electrostatic retention onto the bead surfaces. Such colloidally stable 3D collections of closely spaced NPs onto the PS core yield highly intense SERS signals.^9,10^ The resulting PS@Ag beads (Figure 1b and Figure S2) were then redispersed into a cetyltrimethylammonium bromide (CTAB) aqueous solution below the critical micelle concentration (CMC) before the direct growth of the outer ZIF-8 shell. Here, CTAB molecules have two important effects. First the CTAB acts as a bridge between the PS@Ag beads and the ZIF-8 precursors by coating the AgNPs on the PS surface, thus allowing the growth of crystals that completely encapsulate the PS@Ag beads. Otherwise, the formation of MOF crystals without plasmonic properties would be prevalent since the MOF precursors would be free. Second, CTAB concentrations added below critical micelle concentration help to obtain ZIF-8 crystals in desired sizes and monodispersity. However, the natural crystal morphology of ZIF-8 changes from truncated rhombic dodecahedron to cubic morphology, as the hydrophobic tail of CTAB more energetically and selectively suppresses the {100} faces of growing crystals.^33^ This change can be easily observed with the representative SEM image in Figure 1d.

For the preparation of the composites, CTAB-modified PS@Ag beads were subsequently combined, under stirring, with aqueous solutions of 2-methylimidazole (mIm) and zinc acetate to yield PS@Ag@ZIF-8 particles of 847 ± 69 nm size (Figure 1c,d and Figure S2). ζ potential measurements reveal that ZIF-8-coated PS@Ag beads have a highly positive surface charge; however, there is a decrease in surface charge due to the linker, mIm, when compared to bare PS@Ag (Figure 1e). UV–vis spectroscopy was also used to monitor the optical evolution of the composites during the different fabrication steps. As shown in Figure 1f, the accumulation of AgNPs onto the PS surface causes a red-shift of the LSPR of the individual NPs (from ca. 418 to 446 nm) and the appearance of a new broad shoulder at longer wavelengths which is associated with the plasmon coupling of surface-bound and closely spaced Ag NPs. Subsequent coating with ZIF-8 determines a dampening of the plasmonic contribution and its further red-shift, which is also consistent with the NP entrapment within the ZIF-8 shell. Nitrogen physisorption and Brunauer–Emmett–Teller (BET) analysis of the composites show the typical reversible type I isotherm (Figure 1g) as previously observed for pure ZIF-8 crystals. Here, the amount of adsorbed N_2_ rapidly increases at low pressures, indicating the existence of micropores.^34,35^ Similarly, the obtained surface area (*S*_BET_ ≈ 1000 m^2^/g) also falls within the common range reported for pristine ZIF-8 particles (ca. 800–1400 m^2^/g) (Figure S3b).^35^ XRD patterns of PS@Ag@ZIF-8 feature both low angle diffractions from ZIF-8 (2θ = 7.4°, 10.41°, 12.75°) and the different diffraction from Ag (2θ = 38.4°), confirming the crystallinity of the composites (Figure 1h).

**Figure 2:**
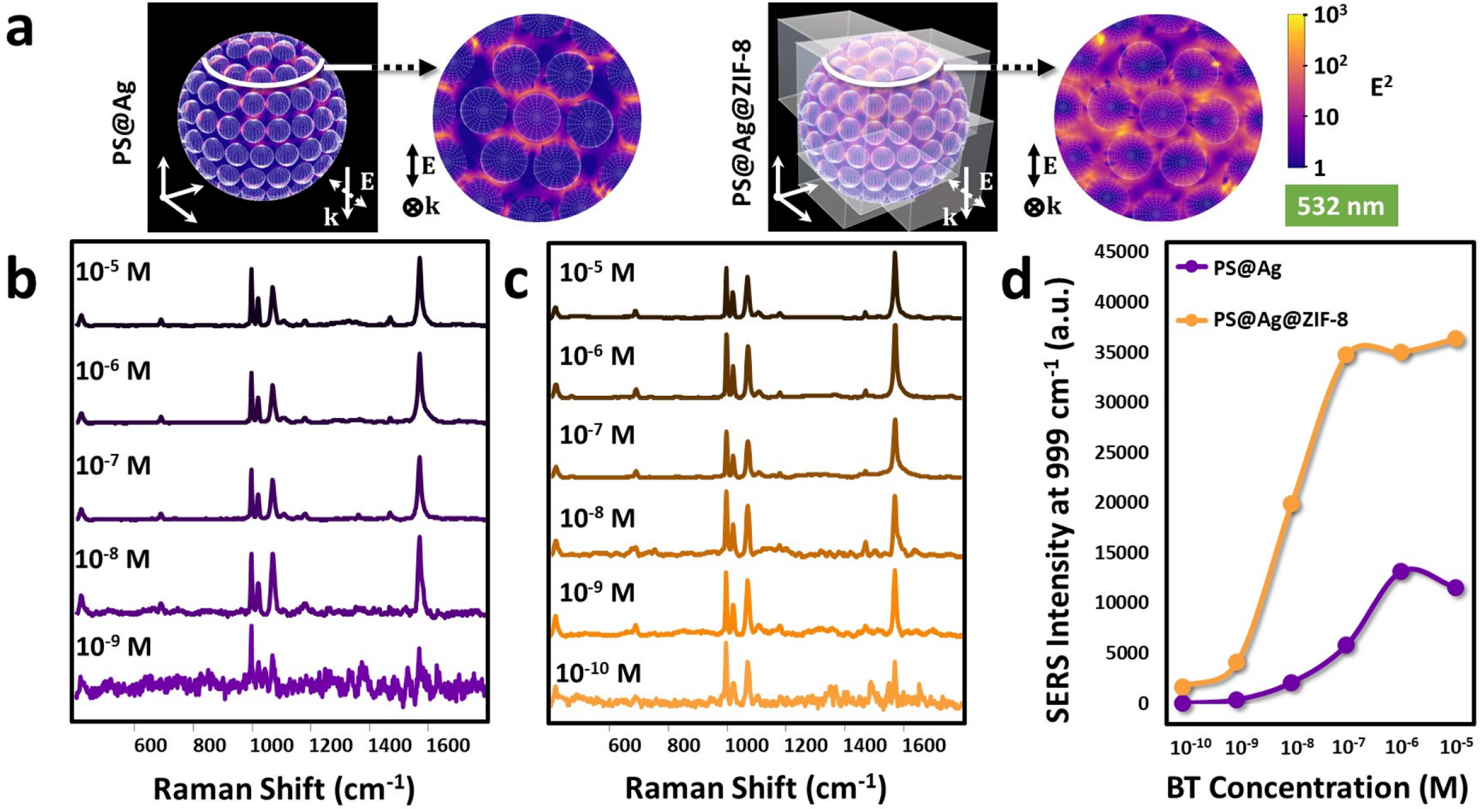
(a) Near field calculations (532 nm) of a model PS@AgNPs before and after coating with ZIF-8. Insets show a top view of the sample within the white border. (b, c) SERS spectra of different concentrations of BT on PS@Ag beads and PS@Ag@ZIF-8 composites. (d) Comparison of absolute SERS intensities at the 999 cm^–1^ band for BT molecules on PS@Ag and PS@Ag@ZIF-8.

Figure 2a shows the near field calculations at 532 nm for a model comprising silver nanoparticles on a polystyrene bead before and after coating with ZIF-8. As expected, the higher enhancements are located at the gaps between the NPs in both samples. Overall, PS@Ag@ZIF-8 shows a higher enhancement than PS@Ag. This increase is ascribed to the deposition of ZIF-8 on the plasmonic structure, which increases the local refractive index and red-shifts the LSPR, thus, producing a better overlap between the excitation light and the LSPR of the material.^36^ To test the SERS enhancing properties of PS@Ag and PS@Ag@ZIF-8 in an aqueous suspension, we used a well-known benzenethiol (BT) as a molecular probe at different concentrations (Figures 2b–d). In a typical experiment, SERS measurements were carried out on equimolar functionalized microbead suspensions using a 532 nm laser and adopting a macro setup configuration. Briefly, the macro setup configuration uses a specialized accessory for the Raman equipment that allows the measurement of liquid samples. In this setup, the laser is focused over a volume, instead of a classical area, and the scattered light is collected. This allows the attainment of quantitatively reliable SERS spectra resulting from the averaged contribution of many beads within the illuminated volume. As can be observed, PS@Ag@ZIF-8 yields detection limits down to 10^–10^ M, 1 order of magnitude lower than bare PS@Ag beads (Figure 2b,c). The absolute intensity of the narrow band at 999 cm^–1^ (ring breathing mode)^37^ is plotted in Figure 2d as a function of BT concentration. Moreover, the absolute intensity at the plateau ([BT] > 10^–7^ M) for PS@Ag@ZIF-8 is ca. 4-fold larger than for bare PS@Ag beads. These results are due to two factors: the better resonance laser-LSPR, in full agreement with the theoretical calculations that indicate a higher near field intensity and thus greater intensity of the SERS signal (Figure 2a); and the accumulation effect derived from the improved affinity of ZIF-8 for the organic probe. This later increases the local concentration of the analyte close to the plasmonic surface with the subsequent increase in the SERS intensity.^38^

**Figure 3:**
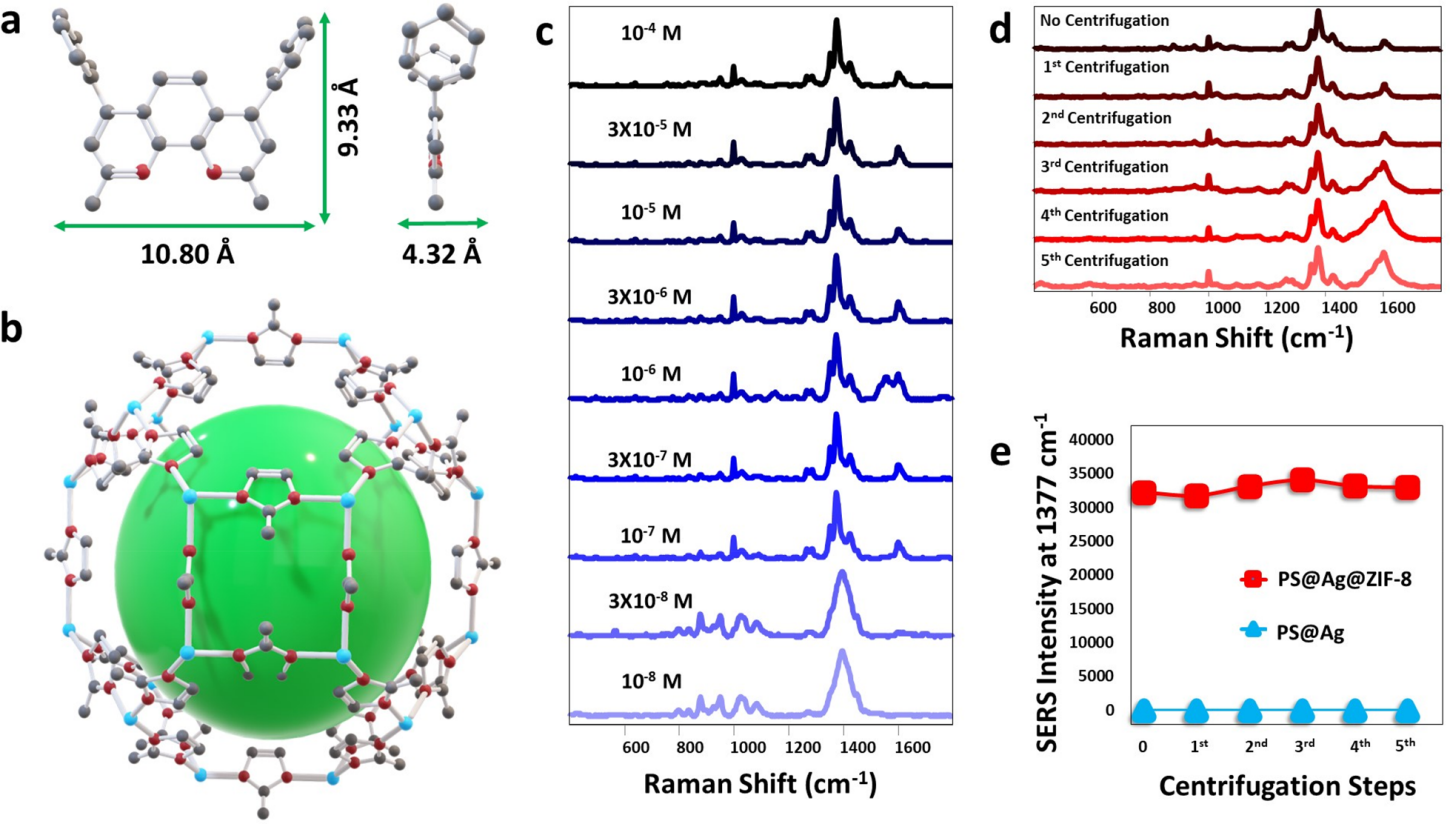
(a) Molecular size of a BC according to the density function theory (DFT) at the B3LYP 6-311G(d, p) level of theory. (b) Schematic representation of the structure of a ZIF-8 crystal. The green sphere represents the pore cavity with nearly 12 Å. Four-membered rings (4MRs) and six-membered rings (6MRs) in the structure have 0.8 and 3.4 Å sizes, respectively. The 6MR can expand to accommodate larger molecules. Color code: C, gray; N, red; and Zn, cyan. Hydrogen was omitted for clarity. (c) SERS spectra of different concentrations of BC adsorbed on PS@Ag@ZIF-8. (d) SERS spectrum of the sediments obtained after several washing cycles. (e) Comparison between the SERS intensities of BC (band at 1377 cm^–1^) on PS@Ag@ZIF-8 and PS@Ag after the same washing samples.

Bathocuproine (BC) has been extensively used as a highly specific ligand for Cu(I) and Cu(II) with no interferences from other cations being reported.^32,39,40^ BC coordinates, through its nitrogen groups, with copper ions to form the BC_2_—Cu complex (Figure S5a). This reaction induces a change in the electronic spectra generating a new band at 483 nm (Figure S5b). The characterization of BC and BC_2_—Cu with Raman, exciting the samples with a 785 nm laser, shows a clear change in their spectral profile (Figure S5c). In summary, a drop in intensity of the band at 1367 cm^–1^ (NC stretching) of BC is mirrored by an increase of the bands at 1425 (CCH bending) and 1563 cm^–1^ (C=C stretching) for BC_2_—Cu. These changes are specific for copper ions and do not appear when other metals are present in solution (Figure S5d). Notably, the excitation of the same samples with a green laser (532 nm) yields an intense fluorescence for BC_2_—Cu that saturates the detector, a signal of the resonance between the green laser and the complex. Because of this resonance, we use the 532 nm source to maximize the SERS signal in our sensor system. To prepare the optical sensor, BC was loaded onto the PS@Ag@ZIF-8 by immersing the composite in an ethanolic solution of the dye at the desired concentration for 12 h. Notably, calculated dimensions for BC are 10.8 × 9.3 × 4.3 Å (Figure 3a). However, calculated dimensions for ZIF-8 pores are 0.8 and 3.4 Å (Figure 3b),^41−43^ bellow 1.5 Å, as calculated from the nitrogen sorption isotherm (Figure S6). Thus, *a priori*, the penetration of the dye is sterically restricted. However, unlike other porous structures such as silica, carbon, or clays, ZIF-8 exhibits dynamic pore properties^27^ due to the swing motion of the methylimidazole ring,^44^ which allows the MOF to adsorb large molecules particularly when in alcohol solutions and for nonpolar adsorbents.^24,45^ Thus, to ensure the appropriate retention of the dye, the supernatants after centrifugation were studied by UV–vis spectroscopy showing no evidence of the presence of dissolved BC. Conversely, the sediments (i.e., PS@Ag@ZIF-8 loaded samples) were studied with SERS. Figure 3c shows the SERS spectra of PS@Ag@ZIF-8 uploaded with different concentrations of BC. The characteristic dye signal (1367 cm^–1^) can be clearly recognized until concentrations of 10^–7^ M in the initial BC. To test the stability of the retained BC, the sediments were redispersed in water and centrifuged several times. After each centrifugation cycle, the supernatants were monitored by UV–vis and sediments by SERS. Neither BC molecules detected in the supernatants nor a decrease in SERS intensity were observed in the sediments (Figure 3d), demonstrating the stability in water of the sensing element. This behavior contrasts with that of the PS@Ag, which under the same conditions shows no signal of BC absorption (Figure 3e).

**Figure 4:**
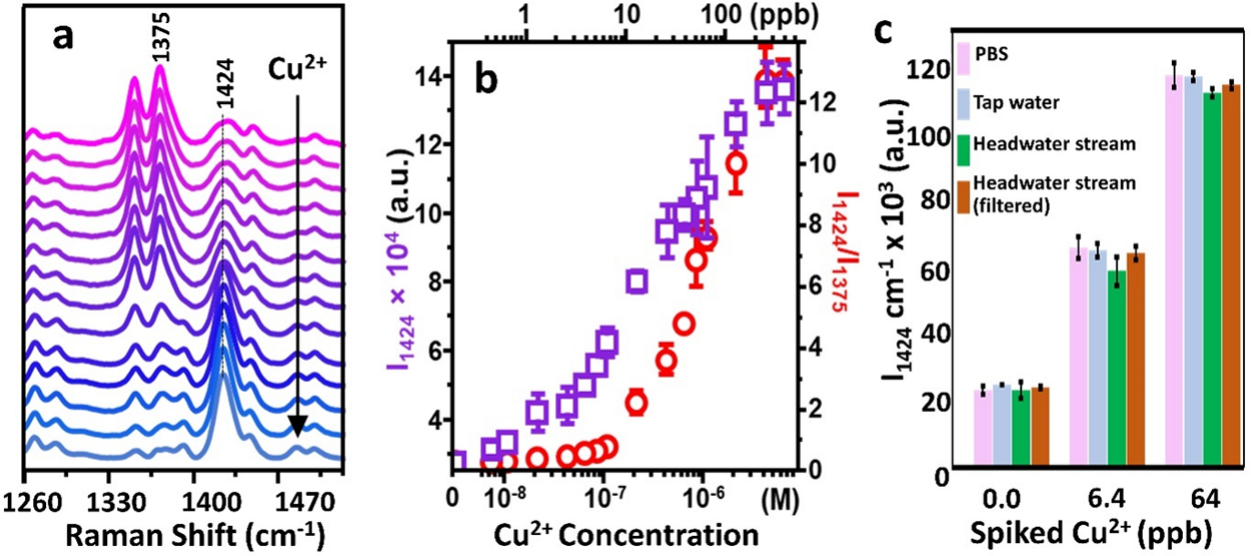
(a) SERS spectra of BC (1 μM) on PS@Ag@ZIF-8 upon immersion into Cu(II) solutions in PBS buffer (pH 7.4) at different copper concentrations (from top to bottom: 0, 0.65, 1.3, 2.5, 3.8, 5.1, 6.4, 12.7, 25.4, 38.1, 50.8, 63.5, 127, and 254 ppb). (b) SERS intensity (*I*_1424_) and intensities ratio (*I*_1424_/*I*_1375_) vs Cu^2+^ concentration in PBS (*N* = 3). (c) SERS intensity *I*_1424_ in pristine PBS, tap water, and headwater stream (unfiltered and filtered) and in the same matrices spiked with Cu(II) at a final concentration of 6.4 and 64 ppb (*N* = 3).

To study the performance of the sensor in the detection of metallic ions, the composite loaded with BC (PS@Ag@ZIF-8-BC) was exposed to several aqueous samples spiked with Cu(II) (Figure 4a). The modification of the SERS fingerprint of BC embedded into the composites upon exposure to a Cu(II) solution is qualitatively analogous to what was observed for the Raman measurements. Furthermore, when PS@Ag@ZIF-8-BC was combined with solutions of Ca^2+^ (1 mM), Cd^2+^ (10 μM), Fe^3+^ (20 μM), Zn^2+^ (40 μM), and Pb^2+^ (10 μM), no distinguishable spectral alterations were detected (Figure S7). It is worth noting that the characteristic vibrational pattern of the BC_2_–Cu^2+^ complex is obtained immediately after the dispersion of the hybrid particles in the sample, indicating the extremely fast diffusion of Cu^2+^ across the ZIF-8 shell to reach the proximity of the inner Ag NP surfaces (i.e., no incubation is required). The Cu^2+^ concentration response of PS@Ag@ZIF-8-BC dispersed in PBS (pH 7.4) is shown in Figure 4a. The data highlight the progressive reshaping of the SERS spectrum to which extent can be quantitatively correlated with the metal ion content. For instance, the bands at 1424 and 1375 cm^–1^, selected as markers of the Cu^2+^ chelation vs free BC, respectively, undergo an intensity increase (1424 cm^–1^) and decrease (1375 cm^–1^) with the increase in copper concentration. Among these two parameters, the intensity of the 1424 cm^–1^ band vs metal ion concentration provides the best outcome, exhibiting an excellent linear response over 2 orders of magnitude (from ca. 250 to 2.5 ppb, which corresponds to ca. 4 μM to 40 nM; *r*^2^ = 0.99) and a limit of detection of ca. 0.6 ppb (10 nM, corresponding to an *I*_1424_ value larger than the blank sample plus 2 times the standard deviation). This level is competitive with techniques such as ICP-MS (0.1 ppb) and far superior to other methods such as ICP (0.3 ppm) or FAAS (0.5 ppm).^46^ On the other hand, the intensity ratio *I*_1424_/*I*_1375_ (Figure 4b) displays a linear response in a narrower Cu^2+^ concentration range (ca. 4 μM to 100 nM, *r*^2^ = 0.970) and a limit of detection of ca. 1.2 ppb (20 nM). It must be stressed that the BC content on PS@Ag@ZIF-8 has been approximately optimized to yield intense SERS signals with a high signal-to-noise ratio, which improves the accuracy and robustness of the SERS response while maintaining the ligand concentration sufficiently low to meet the specific requirements of sensitivity and dynamic linear response for Cu^2+^ detection (Figure S8). Besides, to demonstrate that the composites are fully functional in different environments, we used tap water and freshwater samples, the latter collected at a headwater stream site located in Campus Lagoas Marcosende, Universidade de Vigo (Spain). Figure 4c shows the SERS intensities of the 1424 cm^–1^ BC marker band in pristine waters as well as samples spiked with CuCl_2_ solution to a final concentration of 6.4 or 64 ppb. Remarkably, no significant changes were detected when compared with the response observed in Cu^2+^ buffer solutions.

## Conclusions

In conclusion, we have demonstrated that a nonfunctionalized ion-selective dye such as BC can be absorbed onto a composite material comprising a plasmonic submicrobead coated with ZIF-8. Contrary to the common strategy of using molecules that bind to the plasmonic surface using a functional group such as a thiol group, MOFs allow the direct loading of the chemosensors in the proximity of the metallic surface preserving the sensing characteristics. This strategy increases the sensing capabilities of the SERS technique by allowing the use of information-rich ligands to the target. The resulting optical sensor can detect Cu(II) at the ultratrace level without further processing by means of SERS. Detection limits with this method are superior to those of induced coupled plasma or atomic absorption and comparable with those obtained with induced coupled plasma coupled with a mass detector.

## Experimental Section

### Chemicals

Silver nitrate (99%, AgNO_3_), l-ascorbic acid (99%, AA), sodium citrate tribasic dihydrate (≥98%, CA), magnesium sulfate (≥97%, MgSO_4_), zinc acetate dihydrate (98%, Zn(CH_3_COO)_2_·2H_2_O), 2-methylimidazole (99%, C_4_H_6_N_2_, mIm), PAH (*M*_w_ = 15000 Da), sodium chloride (≥99.5%, NaCl), potassium chloride (≥99%, KCl), sodium phosphate dibasic (≥99%, Na_2_HPO_4_), sodium phosphate monobasic (≥99%, NaH_2_PO_4_), aluminum nitrate nonahydrate (≥98%, Al(NO_3_)3·9H_2_O), cobalt(II) nitrate hexahydrate (98%, Co(NO_3_)_2_·6H_2_O), copper(I) iodide (99.9%, CuI), copper(II) chloride dihydrate (≥99%, CuCl_2_·2H_2_O), iron(II) chloride (98%, FeCl_2_), iron(III) chloride hexahydrate (≥99%, FeCl_3_·6H_2_O), nickel(II) nitrate hexahydrate (≥98.5%, Ni(NO_3_)_2_·6H_2_O), BT (95%), and ethanol (99.5%) were purchased from Sigma-Aldrich. Bathocuproine (98%, BC) was purchased from Fisher Scientific. The polystyrene bead solution (ca. 500 nm, AJ50) was purchased from Ikerlat Polymers. CTAB (≥99%) was purchased from Acros Organics. All reactants were used without further purification. Milli-Q water (18 MΩ cm^–1^) was used in all aqueous solutions, and all the glassware was cleaned with aqua regia before the experiments.

### Synthesis of Silver Nanospheres

Synthesis of Ag colloids was carried out as previously reported.^47^ Briefly, 100 mL of H_2_O was heated until boiling. Then, an aqueous mixture of 100 μL of AA (0.1 M) and 1.364 mL of CA (0.1 M) was added under strong magnetic stirring. After 1 min, another aqueous mixture of 298 μL of AgNO_3_ (0.1 M) and 224 μL of MgSO_4_ (0.1 M) was added (the mixture was previously incubated for 5 min). The solution was left boiling under stirring for another 30 min and left to cool down at room temperature before being centrifuged once (6500 rpm, 15 min). The pellet was redispersed with Milli-Q water to yield a final Ag^0^ concentration of 1.4 mM.

### Deposition of Ag NPs on PS Beads (PS@Ag)

PS of ca. 500 nm diameter were initially coated with positively charge PAH. To this end, 10 mg of PAH was added to 10 mL of NaCl aqueous solution (0.5 M) and sonicated for 30 min. Then, 100 μL of PS bead dispersion (100 mg/mL) was added to 9.9 mL of aqueous PAH solution and stirred at 500 rpm for 30 min. The sample was then subjected to three centrifugation–washing cycles (9000 rpm, 30 min) with Milli-Q water to remove unbound PAH. Finally, the PS@PAH beads were redispersed in 50 mL of Milli-Q water (PS concentration = 0.2 mg/mL). To this sample, 10 mL of the Ag colloids ([Ag0] = 1.4 mM) was added dropwise under sonication. Immediately after, the mixture was stirred (300 rpm) for another 30 min. Finally, the mixture was subjected to three centrifugation–washing cycles (4500 rpm, 15 min) with Milli-Q water to remove unbound Ag NPs. The resulting PS@Ag beads were redispersed in 10 mL of Milli-Q water (PS@Ag concentration = 1 mg/mL).

### Preparation of ZIF-8-Coated PS@Ag Beads (PS@Ag@ZIF-8)

157.9 μL of CTAB aqueous solution (10 mM) was added to 3 mL of PS@Ag suspension (1 mg/mL) and stirred for 30 min for adsorption of CTAB to the plasmonic surface. Then, 3 mL of mIm aqueous solution (1.32 M) was added under stirring (500 rpm) followed, 10 min later, by 3 mL of Zn(CH_3_COO)_2_ aqueous solution (24 mM). The mixture was kept under stirring for 5 more minutes and, subsequently, incubated at room temperature for 3 h. Afterward, the sedimented PS@Ag@ZIF-8 particles were separated from the whitish supernatant and were subjected to two centrifugation–washing cycles (4500 rpm, 5 min) to remove residual ZIF-8 particles in the supernatant. The PS@Ag@ZIF-8 pellet was finally redispersed in 15 mL of Milli-Q water (PS concentration = 0.2 mg/mL).

### Sample Preparation for Raman Analysis

Two aliquots of 500 μL of equimolar ethanolic solutions (2 × 10^–3^ M) of BC and metal cation solutions were combined. Immediately after, the color changed from transparent to vermilion. The solvent was removed by evaporation at 60–65 °C. The residual solids were collected and characterized by Raman spectroscopy.

### Sample Preparation for SERS Analysis

500 μL of PS@Ag@ZIF-8 (0.2 mg/mL) was added to 10 mL of ethanolic solutions of BT or BC at the desired concentration. The mixtures were incubated overnight and then subjected to two centrifugation–washing cycles (4500 rpm, 5 min) with Milli-Q water. BC-loaded PS@Ag@ZIF-8 were finally redispersed in 1 mL of Milli-Q water. An identical protocol was applied for PS@Ag particles. For metal ion detection, 100 μL of BC-loaded PS@Ag@ZIF-8 particles was combined with 1 mL of metal ion solution, at different Cu(II) concentrations, prepared directly in PBS buffer (pH 7.8) or by spiking tap water and freshwater with appropriate aliquots of a CuCl_2_ solution. Similar protocols were applied for the detection of other metal cations. Filtration of the samples, when applied, was performed using a 0.45 μm pore diameter filter paper.

### Theoretical Calculations

Simulations were performed with the Boundary Element Method^48^ using the MNPBEM toolbox.^49^ The silver dielectric constant is from Palik’s handbook.^50^ For the simulation, we considered the MOF as a perfect dielectric, *n* = 2.039. Volume and cross sectional area of the probe molecules were calculated with DFT at the B3LYP 6-311G(d,p) level of theory using Gaussian 16.^51^

### Instrumentation

UV–vis spectroscopy (Agilent Technologies, Cary 8454), TEM (JEOL JEM 1010), and FIB-SEM (Helios NanoLab 600) operating at an acceleration voltage of 100 kV were applied to characterize the optical response and size of the particles. The samples were prepared by drying suspensions on carbon-Formvar-coated 200-mesh copper grids. ζ potential studies were carried out with a Malvern Zetasizer Nano ZS instrument. Physisorption studies were carried out with N_2_ at 77 K using a Belsorp-max apparatus from MicrotracBEL Corporation (Osaka, Japan). Before being analyzed, the samples were outgassed at room temperature for 12 h under a pressure of 0.1 Pa. The BET processing was carried out in the relative pressure range of 0.05–0.25. XRD analysis was carried out with a Siemens D5000 instrument. Raman and SERS spectra were collected in backscattering geometry with a Renishaw inVia Reflex system equipped with a 2D-CCD detector, a Leica confocal microscope, and two excitation sources: a 532 nm frequency doubled Nd:YAG/Nd:YVO4 diode, and a 785 nm NIR diode laser. The 532 nm laser was focused on the colloidal suspension using a macrolens (10 s exposure, 3 accumulations) with power at the sample of 17.1 mW. Normal Raman spectra of BC/metal cation powders were obtained using a 785 nm laser to remove background fluorescence.

## Supporting Information Available

Extra TEM images and UV–vis, Raman, and SERS spectra (PDF)
