# Supplementary material for: Optical Quantification of Metal Ions Using Plasmonic Nanostructured Microbeads Coated with Metal–Organic Frameworks and Ion-Selective Dyes
Source: ACS Nanosci Au. 2023 Mar 6;3(3):222–9. doi: 10.1021/acsnanoscienceau.2c00063 (PMC10288605; doi:10.1021/acsnanoscienceau.2c00063)
Supplement: Supplementary file 1 — ng2c00063_si_001.pdf [file ng2c00063_si_001.pdf]

Supporting information for:

Optical Quantification of Metal Ions using Plasmonic Nanostructured Microbeads Coated with Metal-organic Frameworks and Ion-selective Dyes

Tolga Zorlu[#,†], Begoña Puértolas[†], I. Brian Becerril-Castro[#], Vincenzo Giannini[§,⊥], Miguel A. Correa-Duarte[†,*], Ramon A. Alvarez-Puebla[#,‖,*]

[#]Department of Physical and Inorganic Chemistry – Universitat Rovira i Virgili, Carrer de Marcel·lí Domingo s/n, 43007 Tarragona, Spain

[†]Department of Physical Chemistry, Center for Biomedical Research (CINBIO), Southern Galicia Institute of Health Research (IISGS) and Biomedical Research Networking Center for Mental Health (CIBERSAM), Universidade de Vigo, 36310 Vigo, Spain

[§]Technology Innovation Institute, Masdar City, 9639 Abu Dhabi, United Arab Emirates

[⊥]Centre of Excellence ENSEMBLE3 sp. z o.o., Wolczynska 133, 01-919 Warsaw, Poland

[‖]ICREA, Passeig Lluís Companys 23, 08010 Barcelona, Spain

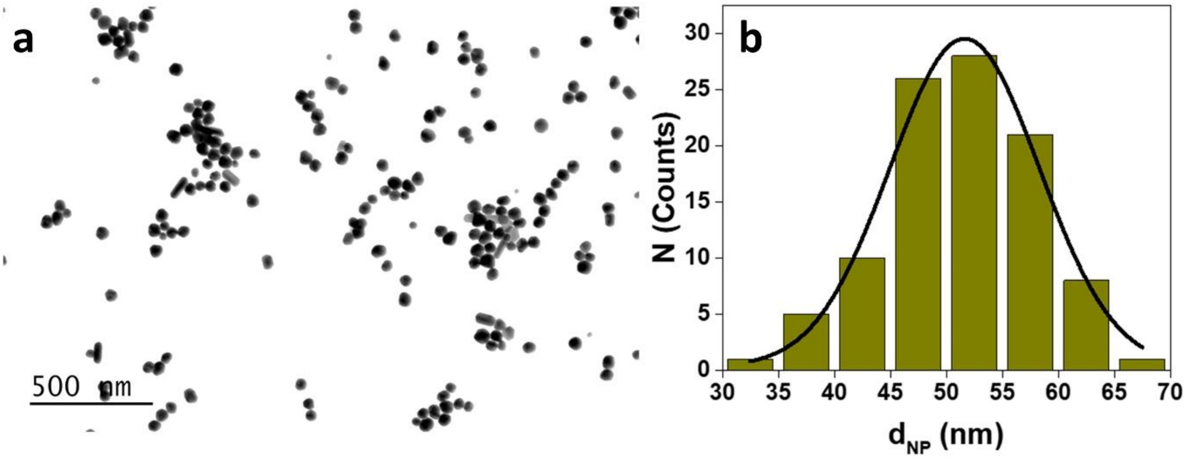

Figure S1. Ag NPs: **(a)** representative TEM image, and **(b)** histogram of the silver NPs (51 ± 6 nm).

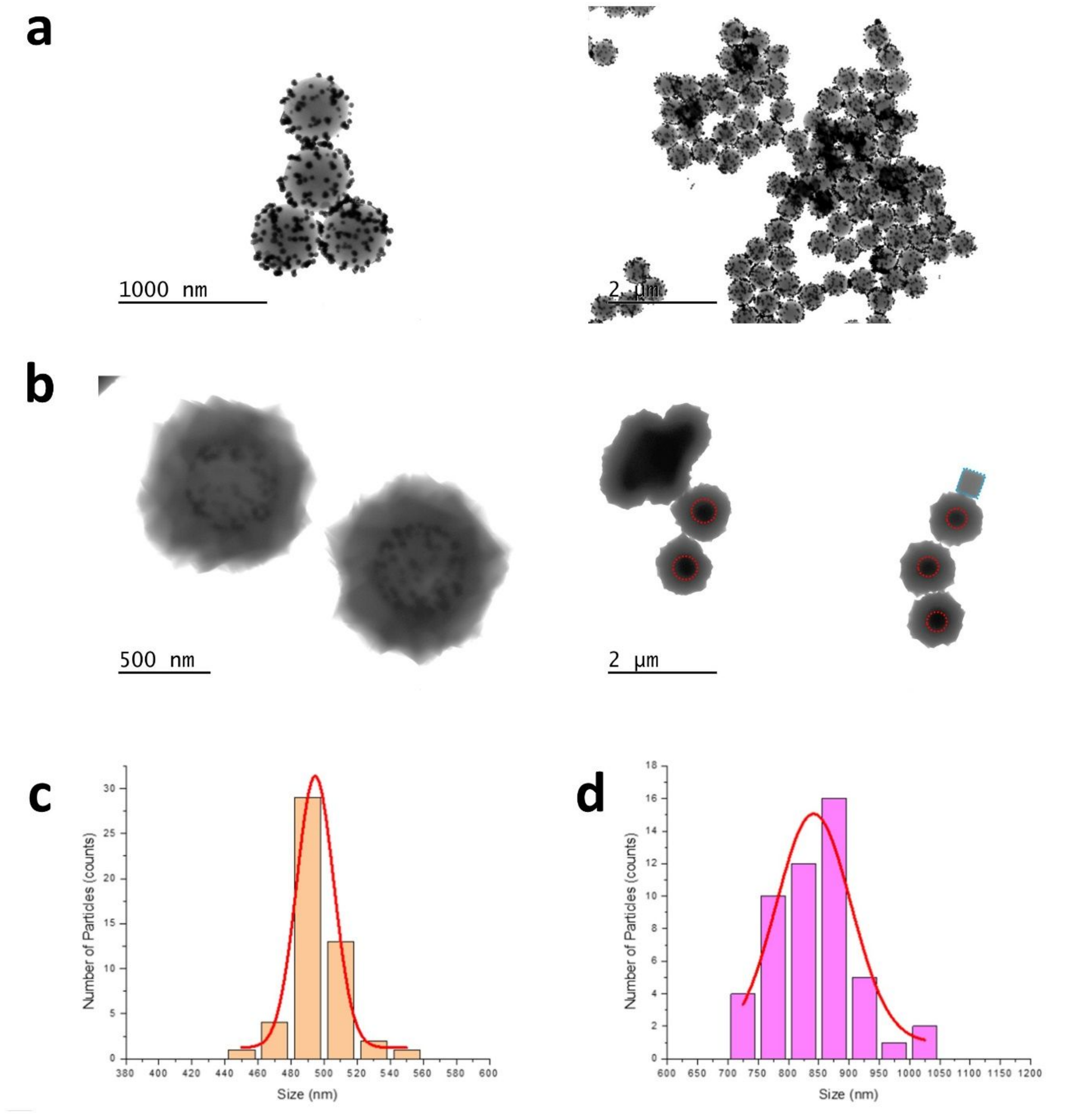

Figure S2. **(a, b)** Additional TEM images of PS@Ag beads and PS@Ag@ZIF-8 composites, respectively. Red circle and blue square in **(b)** represent the PS bead and free ZIF-8 crystal, respectively. **(c, d)** Histograms of PS beads and PS@Ag@ZIF-8 composite diameters, respectively.

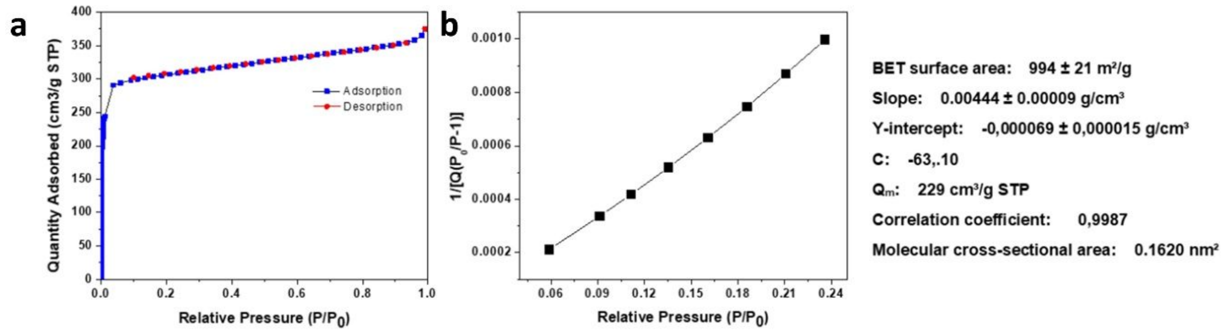

Figure S3. **(a)** Nitrogen adsorption–desorption isotherms of PS@Ag@ZIF-8 composites and **(b)** BET surface area plot.

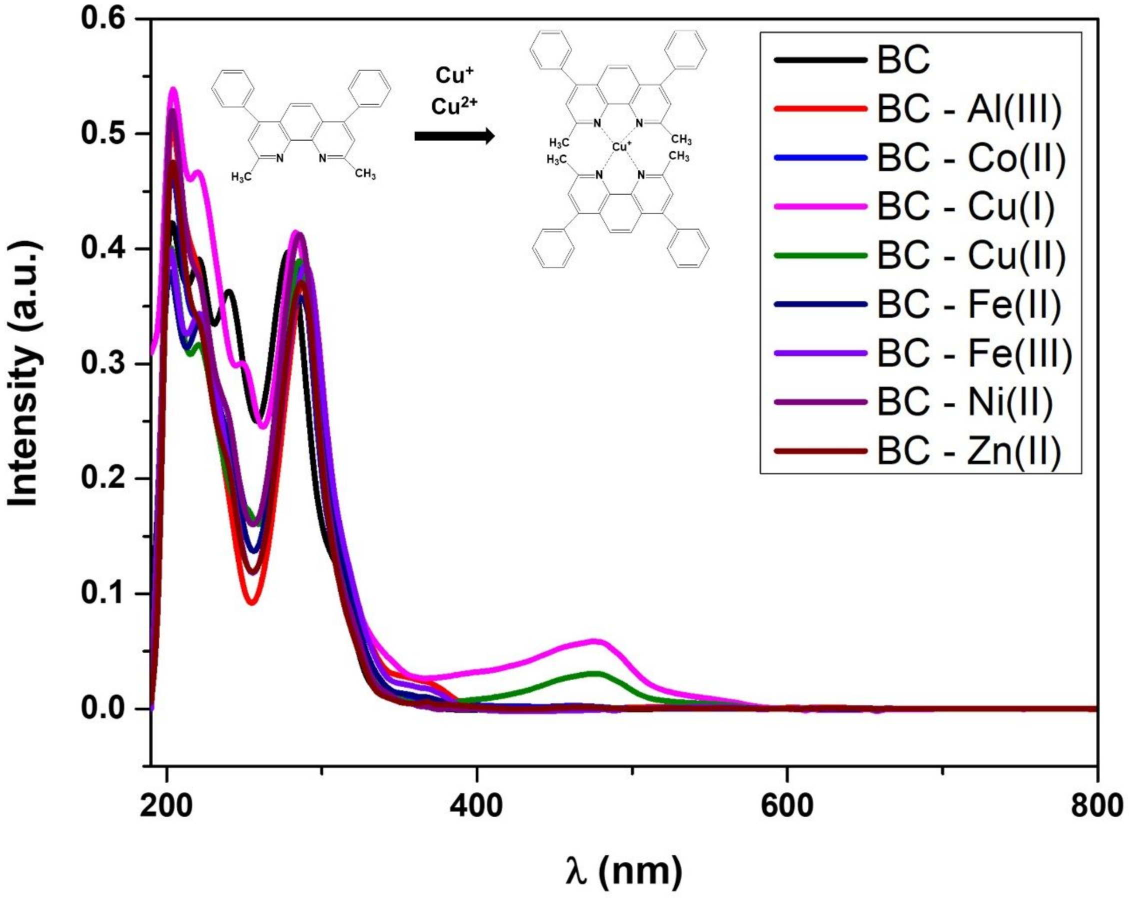

Figure S4. Absorption spectra of BC ethanolic solutions (10 μM) upon addition of different metal ions (final concentration = 5 μM).

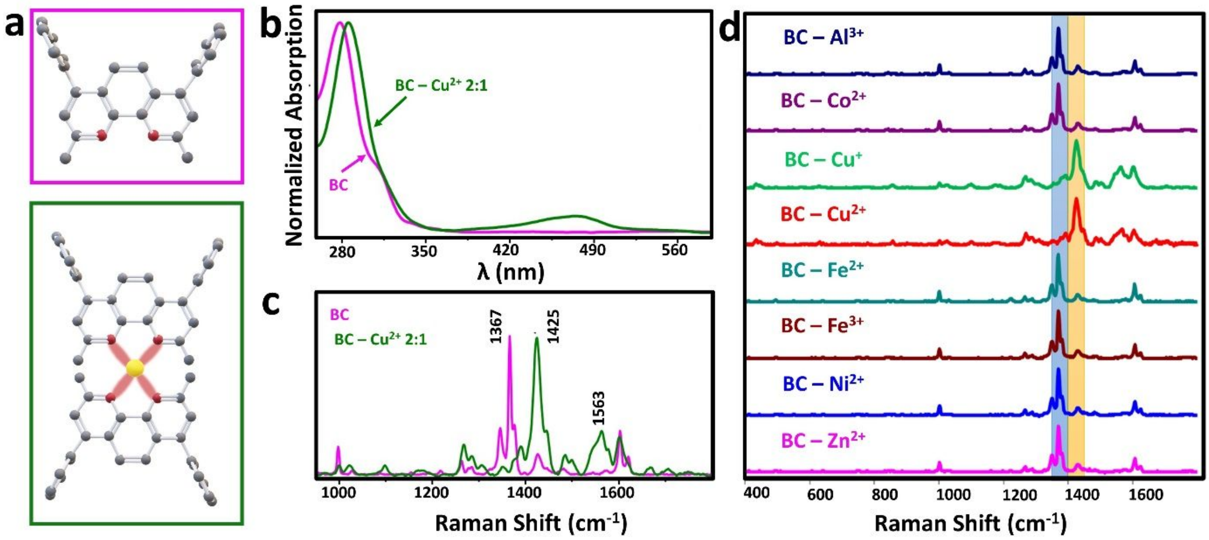

Figure S5. (a) Molecular structures of BC (above), and BC_2-Cu^{2+} complex (below). Colour code: C: grey, N: red, and Cu: yellow. Reddish stripes indicate the coordination bonds between N and Cu atoms. Hydrogen atoms were omitted for clarity. (b) Absorption spectra of BC in ethanol (10 μM) before and after the addition of Cu^{2+} (ligand/metal molar ratio = 2:1). (c) Raman spectra of the corresponding solids (excitation wavelength = 785 nm). (d) Raman spectra of the solids were obtained by mixing ethanolic solutions of BC and metal ions at a 2:1 molar ratio. Upon solvent evaporation, the remaining powder was collected and interrogated by Raman. Excitation wavelength = 785 nm.

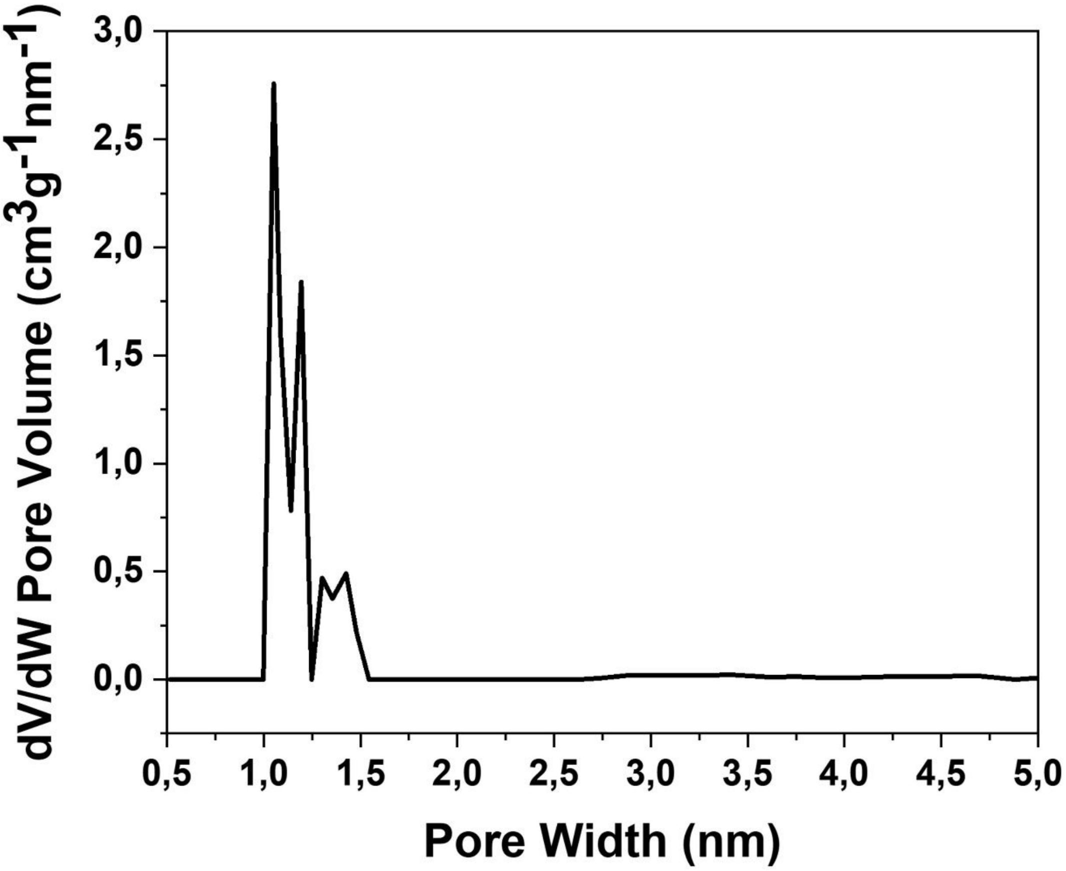

Figure S6. Pore size distribution of PS@Ag@ZIF-8 composites.

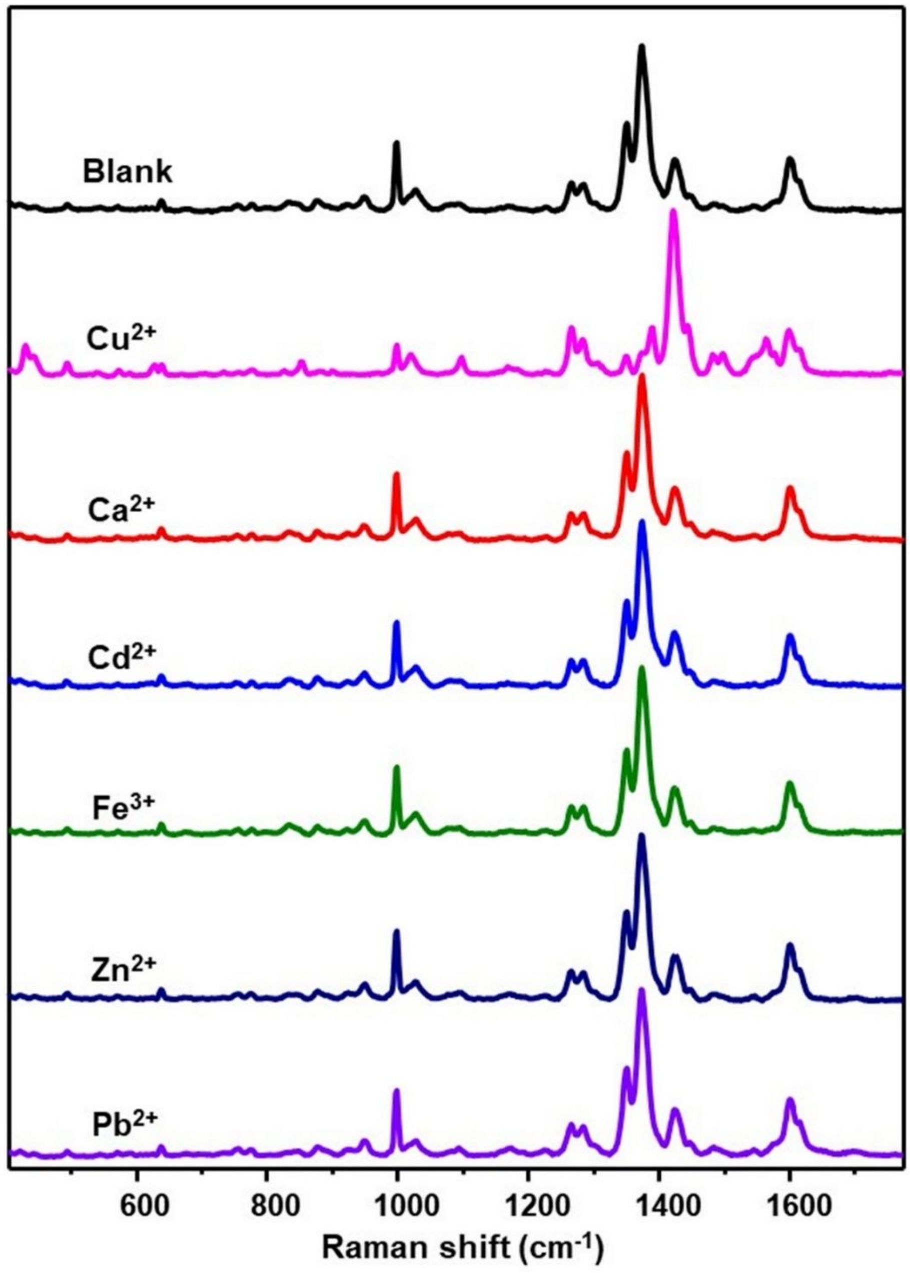

Figure S7. SERS spectra of BC 1 μM on PS@Ag@ZIF-8 (blank) and in the presence of Cu^{2+} (2 μM), Ca^{2+} (1 mM), Cd^{2+} (10 μM), Fe^{3+} (20 μM), Zn^{2+} (40 μM) and Pb^{2+} (10 μM). Excitation wavelength = 532 nm.

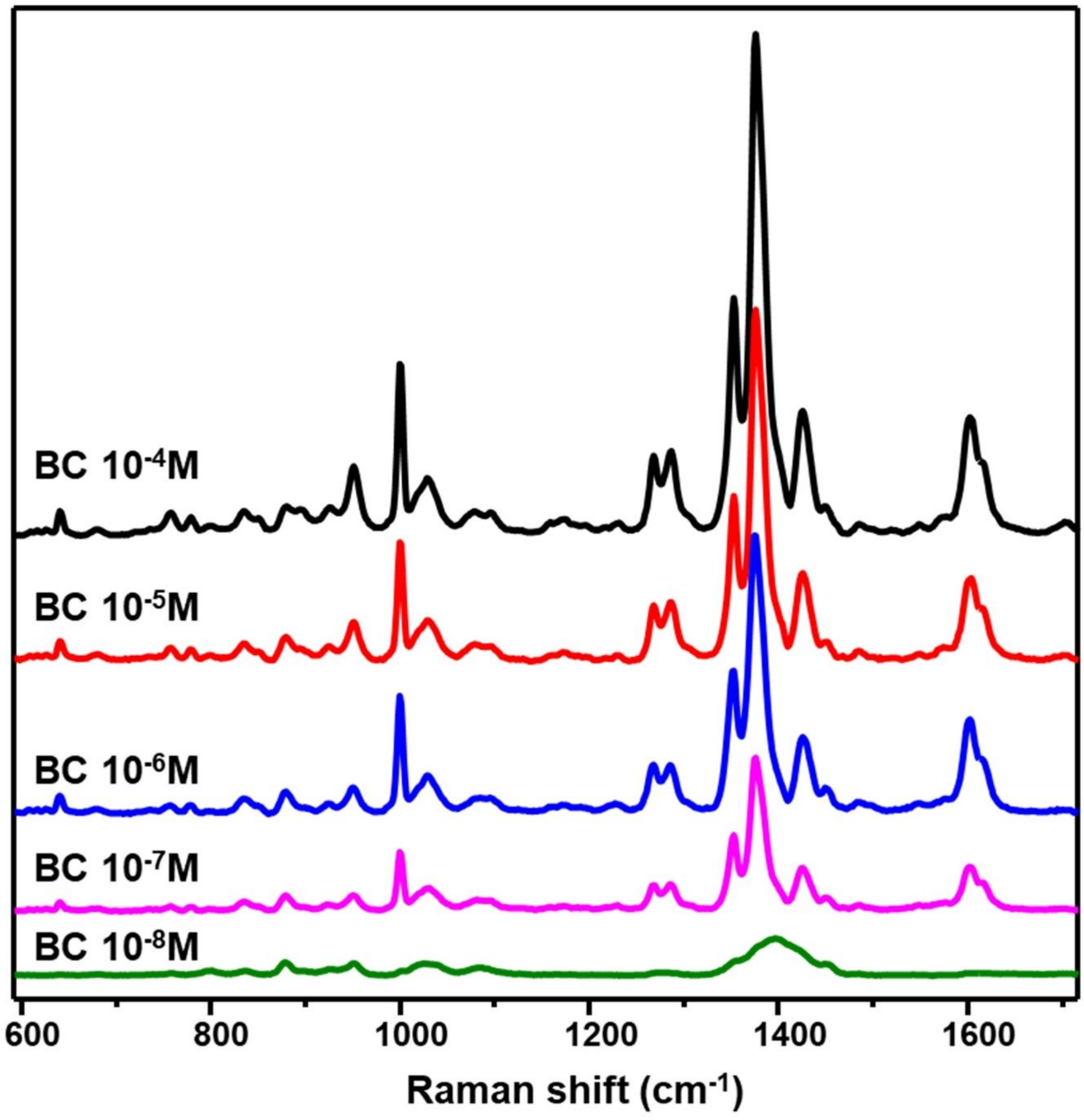

Figure S8. SERS spectra of different concentrations of BC on PS@Ag@ZIF-8 in PBS buffer (pH 7.4) (excitation wavelength = 532 nm).
